# Book Review: Understanding Dialogue: Language Use and Social Interaction

**DOI:** 10.3389/fpsyg.2021.736276

**Published:** 2021-09-01

**Authors:** Keshu Xiang

**Affiliations:** School of Foreign Languages, Shanghai Jiao Tong University, Shanghai, China

**Keywords:** dialogue, language production, linguistic representation, alignment, workspace framework, psycholinguistics

We have dialogue with other people every day. Dialogue is so common to our daily life. However, we know very little about the cognitive mechanism of dialogue. *Understanding Dialogue: Language Use and Social Interaction*, written by Pickering and Garrod, is a timely book offering profound insights into the cognitive and social mechanisms of dialogue (Pickering and Garrod, 2021). Consisting of an introductory chapter, a conclusion chapter and four main parts in another ten chapters, this book presents the state-of-the-art research on the complex world of dialogue.

The introductory chapter (Chapter 1) presents the challenge of dialogue. Based on Bratman's (1992) notion of shared cooperative activity, Pickering and Garrod argue that dialogue is a cooperative joint activity, in which interlocutors cooperate with each other under a shared workspace framework. The first main part (Chapters 2–5) introduces the shared workspace framework for cooperative joint activities. In Chapter 2, Pickering and Garrod first introduce the cooperative joint activities to highlight problems that interrogators have to confront to understand dialogue and then they put forward the shared workspace framework for such activities. Chapter 3 explores how individuals execute, understand and control joint activities under the shared workspace framework. In Chapter 4, Pickering and Garrod apply the shared workspace framework to dialogue and discuss four characteristics of the dialogue system (i.e., alignment, simulation, prediction, and synchrony). Alignment is “a property of the relationship between the interlocutors' representations” (p. 85). When the interlocutors are fully aligned, they will share similar representations and behave similarly to each other (Pickering and Garrod, 2004). Prediction is crucial for a smooth dialogue. Interlocutors use prediction to control their initiation of turns in a dialogue. Simulation and synchrony make alignment and prediction work in the right trace under the shared workspace framework. Chapter 5 explains how interlocutors engage in dialogue under the shared workspace framework. The second main part (Chapters 6–7) primarily focuses on alignment during dialogue. Chapter 6 introduces four kinds of alignment (i.e., focal alignment, global alignment, linguistic alignment and dialogue model alignment). Chapter 7 turns to discuss the alignment of the dialogue models: the situation model and the dialogue game model. The third main part (Chapters 8–9) explains how interlocutors use the shared workspace framework efficiently. To use the shared workspace framework efficiently, interlocutors should “say just enough (Chapters 8)” and “speak in good time (Chapters 9).” To say just enough in the right time, interlocutors should actively participate in the dialogue, make prediction and provide brief but sufficient feedback timely. The fourth main part (Chapters 10–11) extends the shared workspace framework to multi-party conversation as well as monolog and explains how to integrate it with cultural institutions and cultural artifacts. This book comes to the conclusion chapter (Chapter 12) after discussing the four main parts mentioned above and once again Pickering and Garrod emphasize the importance and challenge of dialogue.

In the past, just as Levelt (1989) pointed out “language production is the stepchild of psycholinguistics” (preface), language production, especially dialogue, has received much less attention than language comprehension. Although some works such as the classical works of Levelt published in 1989 have discussed the issue of individual representation in speaking, they seldom touched on the problem of “more than one individual's representations” in dialogue. “Drawing on psychological, linguistic, philosophical and sociological research,” Pickering and Garrod's book makes up this gap and provides readers with the scientific analysis of dialogue.

Overall, this book provides readers with an innovative research on dialogue. It is very informative, well-organized and reader-friendly in writing. Firstly, as an innovative attempt to investigate the cognitive and social mechanisms of dialogue, the core contribution of this book perhaps lies in proposing the shared workspace framework for dialogue. At the very beginning, it emphasizes that the dialogue should be analyzed under a united system. With this systems-based approach, the relationships between individual cognitive representations can be investigated. Then, to make the shared workspace framework successfully work, the authors innovatively propose four key characteristics of the dialogue system: alignment, simulation, prediction and synchrony. Under the shared workspace framework, Pickering and Garrod provide people with a convincing and practical way to investigate dialogue.

The shared workspace framework for dialogue proposed by Pickering and Garrod resembles the classical modular model of language production put forward by Levelt (1989) in some respects. The modular model assumes that speech production is modular and language production mainly consists of four important components: conceptualization, formulation, articulation and self-monitoring. This model holds the view that each module operates independently and possesses its own unique input and output. That is, the representation of different linguistic levels is independent of each other. Similarly, in the shared workspace framework for dialogue, Pickering and Garrod assume that there are three linguistic levels concerning with meaning, grammar and sound, which are corresponding to semantics, syntax and phonology, respectively. As Levelt's model, the shared workspace framework also assumes an independent representation of different linguistic levels. Importantly, Pickering and Garrod's model outperforms Levelt's (1989) model, in that it extends the scope of representations from that of isolated speaker or one isolated listener to both sides of interlocutors through alignment under the shared workspace framework.

Secondly, this book is well-organized in structure. Four main parts covering different aspects of dialogue make the whole book into a united, cohesive, and coherent union. Judging from the lay out of this book, it is not difficult to find that the whole book is laid out surrounding the shared workspace framework for dialogue. Each chapter of this book is more or less involving with the shared workspace framework for dialogue and the content in each chapter manifests an incremental style. That is, the former chapter lays the foundation for the latter, and the latter is the deepening of the former. All chapters together make the whole book a united union.

Thirdly, this book is reader-friendly in writing. Almost all chapters in this book contain a beginning section to briefly remind readers what have been discussed in the previous chapter and a conclusion section to conclude the main content of the whole chapter. The text contains very few acronyms, formulas, or concepts that are hard to follow. Instead, it provides many examples, cases, vivid graphics, and a brief glossary to help readers get through the whole book easily. Therefore, it should be readable by all researchers working in the fields of linguistics, psychology and cognitive linguistics.

Despite the above strengths, this book is not without its weaknesses. Although this book provides a well-established theory on dialogue, it does not touch upon the possibility of conversational form among non-human animals or the case of conversational deficits. It is generally accepted that non-human animals have no language (Berwick and Chomsky, 2016). However, it doesn't mean that non-human animals have no way to make their own “dialogues” (Hockett, 1959). For example, the honeybee can use the waggle dance to tell other bees about the location and distance of a food source. Can such kinds of communication of non-human animals be treated as a type of dialogue? If the answer is yes, and then in what forms does the dialogue of non-human animals be similar to or different from human beings' dialogue? Moreover, for the issues of dialogue among disabled people with a visual or hearing impairment, how the dialogue is going on? Can the shared workspace framework for dialogue still work for this kind of people? Furthermore, by concentrating exclusively on first language (L1), Pickering and Garrod do not take into consideration the difficulties in conversational communication in which both second language (L2) and third language (L3) are involved. Thus, it is not yet clear whether the shared workspace framework will work for dialogue beyond L1. Therefore, in the future version of this book, a comprehensive consideration to dialogue concerning non-human animals or the case of conversational deficits as well as L2 or L3 is expected.

Furthermore, the book has some questionable points in the presentation. This book is concentrated on dialogue. However, it takes two whole chapters (Chapter 2 and 3) to introduce and discuss the cooperative joint activity and the shared workspace framework, which are not highly and directly relevant to dialogue at this moment. Admittedly, this two chapters lay a solid foundation for the latter parts, especially for the application of the shared workspace framework to dialogue. However, as a reader who is more concerned about the issues of dialogue, I would rather the authors contract Chapter 2 and 3 into a short chapter and provide readers with the issues of dialogue earlier. Moreover, there are some small flaws on the details of this book. For example, Figure 4.1 in Chapter 4 is wrongly written as Figure 4.2 in page 90 and 111 in Chapter 5 and 6 respectively.

To conclude, notwithstanding these minor deficits, this book contributes substantially to the domain of language production. Just as the well-known scholar Hartsuiker pointed out in the preface of this book, “This book is a milestone in our understanding of dialogue that will influence the field for decades,” this book has made a great breakthrough in the study of dialogue and will have a profound impact on the study of language production.

## Author Contributions

The author confirms being the sole contributor of this work and has approved it for publication.

## Conflict of Interest

The author declares that the research was conducted in the absence of any commercial or financial relationships that could be construed as a potential conflict of interest.

## Publisher's Note

All claims expressed in this article are solely those of the authors and do not necessarily represent those of their affiliated organizations, or those of the publisher, the editors and the reviewers. Any product that may be evaluated in this article, or claim that may be made by its manufacturer, is not guaranteed or endorsed by the publisher.
